# Plasticity of opsin gene expression in the adult red shiner (*Cyprinella lutrensis*) in response to turbid habitats

**DOI:** 10.1371/journal.pone.0215376

**Published:** 2019-04-12

**Authors:** Chia-Hao Chang, Hong Young Yan

**Affiliations:** 1 Department of Life Science, Tunghai University, Taichung City, Taiwan; 2 Center for Ecology and Environment, Tunghai University, Taichung City, Taiwan; 3 National Museum of Marine Biology & Aquarium, Checheng, Pingtung, Taiwan; University of Sussex, UNITED KINGDOM

## Abstract

Vision is very important to fish as it is required for foraging food, fighting competitors, fleeing from predators, and finding potential mates. Vertebrates express opsin genes in photoreceptor cells to receive visual signals, and the variety of light levels in aquatic habits has driven fish to evolve multiple opsin genes with expression profiles that are highly plastic. In this study, red shiners (*Cyprinella lutrensis*) were exposed to four water turbidity treatments and their opsin genes were cloned to elucidate how opsin gene expression could be modulated by ambient light conditions. Opsin gene cloning revealed that these fish have single *RH1*, *SWS1*, *SWS2* and *LWS* genes and two *RH2* genes. Phylogenetic analysis also indicated that these two *RH2* opsin genes–*RH2A* and *RH2B* –are in-paralogous. Using quantitative PCR, we found evidence that opsin expression is plastic in adults. Elevated proportional expression of *LWS* in the cone under ambient light and turbid treatment indicated that the red shiner’s visual spectrum displays a red shift in response to increased turbidity.

## Introduction

Fish make up the largest and most diverse group of vertebrates; some species have successfully adapted to a variety of physiologically challenging habitats that range from freshwater to seawater, cold polar regions to hot desert climates, and the Mariana Trench to high mountain lakes. With rare exceptions, such as eyeless cave-dwelling species, vision is necessary for fish to forage for food, fight competitors, flee from predators, and find potential mates [1].

The lighting environment is characterized by the spectral composition of light (intensity at each wavelength), the total intensity of light, the transmission properties of the medium (such as air or water), the locations exposed to light, and the surrounding visual background [2]. Compared to terrestrial habitats, aquatic environments have much more diverse lighting environments. This variability arises at least partially because water itself selectively absorbs light–blue light can penetrate deeper than other wavelengths–and the substances suspended or dissolved in water also absorb and/or scatter light. As such, the contents of total of dissolved organics could cause a shift in the dominant light wavelength from blue to longer wavelengths when transitioning from marine to fresh water [3, 4]. Rivers in the Amazon Basin can be classified into three types–white, black, and clear–with each water type exhibiting distinct underwater irradiance based on the amount of inorganic suspended particles and/or dissolved humic substances [5].

In order to receive visual signals, vertebrates rely on visual pigments located in light-accessible outer segments of photoreceptor cells. These pigments contain vitamin A-derived choromophores, including vitamin A1 (retinal) or A2 (3,4-dehydroretinal), that are linked to opsin proteins, which are G protein-coupled receptors. Compositional differences in either the type of choromophore or opsin gene product may alter the maximal absorbance wavelength (λ_max_) of visual pigments [6]. Vertebrates have two kinds of photoreceptor cells, rod cells and cone cells, and five families of opsin genes. The rod cell is used for scotopic vision and only expresses one family of opsin, rhodopsin (*RH1*). The cone cell is used for photopic vision and expresses the other four families of opsins: short-wavelength sensitive 1 (*SWS1*, λ_max_ ranges from UV to violet), short-wavelength sensitive 2 (*SWS2*, λ_max_ ranges from violet to blue), medium-wavelength sensitive (*RH2*, λ_max_ green; similar to *RH1*), and long-wavelength sensitive (*LWS*, λ_max_ red) [6].

Living in aquatic habitats and facing variable lighting environments, fishes have evolved three genetic features for perceiving color in ambient light conditions: opsin gene duplication, mutations in opsin genes, and plasticity in opsin expression [7–9]. Some fish have more than one locus for each cone-expressed opsin gene, and these duplicated opsins may have different λ_max_ values. For example, zebrafish (*Danio rerio*) were shown to have two *LWS* and four *RH2* paralogs; the λ_max_ values of *LWS-1* and *LWS-2* were 558 and 548 nm, while those of *RH2-1* to *RH2-4* were 467, 476, 488 and 505 nm, respectively [10]. Medaka (*Oryzias latipes*) also has two *LWS* paralogs, plus three *RH2* and two *SWS2* paralogs. The λ_max_ values of medaka *LWS-A* and *LWS-B* were reported to be 561 and 562 nm; *RH2-A*, *RH2-B* and *RH2-C* were 452, 516 and 492 nm; and *SWS2-A* and *SWS-2B* were 439 and 405 nm, respectively [11].

Opsin gene mutations that cause substitutions in amino acids, especially those that interact with the chromophore, can greatly affect the λ_max_ value of the visual pigment [12]; therefore, some adaptations may be based on selection for certain residues at critical amino acid-encoding positions of opsin genes. For example, in Lake Tanganyika cichlids, alanine or serine at residue 292 of *RH1* was highly correlated with the preference for shallow or deep water [13]. Additionally, because lighting environments are distinctly different between the Baltic and North Seas, the Baltic Sea populations of sand goby (*Pomatoschistus minutus*) exhibited significant diversifying selection on the *RH1* gene, but the North Sea ones exhibited stabilizing selection on the same locus [14]. Moreover, selection on the *LWS* gene of Lake Victoria cichlids and guppies (*Poecilia reticulata*) not only helped the species adapt to the environment but also resulted in the differentiation of mating preference [15–18]

Although opsin gene duplications and mutations have equipped fish with a variety of opsins, it takes time for a population to acclimate to both through evolutionary events; these evolutionary adjustments cannot immediately enable an individual fish to adjust its visual sensitivity to new surroundings. Many fishes have been found to rely on the plasticity of opsin gene expression to cope with novel or varied lighting environments due to transitory events, such as life history transitions, movement between habitats, or seasonal changes in habitat. The salmonid fishes exhibited a time-dependent opsin expression switch from *SWS1* to *SWS2*, which brought about a red-shifted detection profile in cone cells [19] in preparation for ocean migration. A similar red-shifted detection pattern in cone cells was also observed in milkfish (*Chanos chanos*) when moving from nursery waters, such as lagoons and estuarine areas, into to the ocean after reaching the juvenile stage [20, 21]. On the other hand, upstream migration of both the Japanese eel (*Anguilla japonica*) and giant mottled eel (*A*. *marmorata*) lead to an ontogenetic blue shift in rod cells [22, 23]. Besides ontogenetic changes in opsin expression patterns, some fishes exhibit plasticity in opsin expression in their early life stage or throughout their lifetime. Opsin expression plasticity during the development of both the guppy and Nile tilapia (*Oreochromis niloticus*) has a profound effect on adult opsin expression profile [24, 25]. However, the bluefin killifish (*Lucania goodei*) displays plasticity in opsin expression as an adult; it can quickly modify cone opsin expression level in response to exposure to clear spring waters or tannic acid-stained swamp waters within a few days [26, 27]. Moreover, it has also been demonstrated that the plasticity that some adult African and Nicaraguan cichlids and damselfishes exhibit in opsin expression correlates with variations in lighting environments [28–30].

Red shiners (*Cyprinella lutrensis*) are small-bodied cyprinids (> 90 mm) that are widely distributed over the Great Plains of the United States. These fish can tolerate extreme environmental conditions–such as low oxygen, high acidity, varied temperature, and turbidity–better than other North American cyprinids [31, 32]. Therefore, red shiners are found in a variety of habitats, ranging from very clear to very turbid rivers [33, 34]. The shiners (*Cyprinella* spp.) have a special crevice spawning behavior. During the breeding season, males express sex-limited nuptial coloration and compete with each other to establish territories. The territorial male expels intruding males through threat displays or ‘mock battles’ to protect spawning substrates (e.g. rocks and twigs), and it courts a female by circling her [35, 36].

Living in various habitats with a wide range of turbidity, red shiners encounter distinct lighting environments, and it has been demonstrated that the fish respond to turbidity through phenotypic plasticity in eye size and visual signals. Red shiners have larger eyes and more intense nuptial coloration in turbid water [37, 38], which demonstrates the possibility that their color perception is plastic. However, it has not been reported whether red shiners exhibit plasticity in opsin gene expression. In light of the observation that eye size and nuptial coloration is positively correlated with turbidity, we hypothesized that the red shiner would also vary its opsin expression profile when housed in various water turbidities. We tested this “turbidity dependent opsin expression” hypothesis by cloning the opsin genes and quantifying the expression level of each cone opsin in the red shiner when fish were exposed to various turbidity conditions.

## Materials and methods

### Subjects

Red shiners (*Cyprinella lutrensis*), ranging from 35 to 50 mm in standard length (SL), were purchased from an aquarium store in Taipei City and transported to the Marine Research Station, Academia Sinica, where they were housed in glass aquaria (90 cm Length x 40cm Width x 40 cm Height) at 25–30°C with a 12L:12D photoperiod for at least one month before being moved to four experimental aquaria (45 cm Length x 43 cm Width x 40 cm Height). Fish were fed *ad libitum* with artificial fish feed and frozen *Artemia* twice a day. All experiments were performed and specimens handled with approval (RFiZOOHY20060701) from the Institutional Animal Care and Use Committee (IACUC) of Academia Sinica.

### Experimental setup

Kaolin (Sigma, USA), which consists of clay particles with high surface area that facilitates suspension, was added to tap water to create turbid conditions, 50, 100, and 200 nephelometric turbidity units (NTU); tap water was assumed to be 0 NTU. The absorbance spectra of these four turbid conditions were measured every 30 nm from 400 to 700 nm by a spectrophotometer (VersaMax ELISA Plate Reader, Molecular Devices Corp., USA) with distilled water as the reference. Each turbid sample was measured with three replicates. The four different NTU levels were set up in individual aquaria, each holding 20 red shiners. Air was pumped into each experimental aquarium, not only to provide oxygen, but also to keep the kaolin particles suspended. The light source for each aquarium was a MASTER TL5 HE 28W/865, PHILIPS, which was placed approximately 28 cm above the water’s surface; the photoperiod was set to 12L:12D. The spectrum of the light source is available on the PHILIPS website (http://www.lighting.philips.com/main/prof/conventional-lamps-and-tubes/fluorescent-lamps-and-starters/tl5/master-tl5-high-efficiency/927926586518_EU/product) and mainly ranges from 400 nm to 700 nm. The ambient light spectra of 0 NTU and 200 NTU experimental aquaria were measured using an AvaSpec Micro (Avantes, Apeldoorn, Netherlands). The light in the water was measured upwelling at 8 cm depth. The relative spectral irradiance was smoothed by a factor of 30 using a simple moving average, and then normalized to where 100% indicated the intensity at 550 nm. The red shiners were fed twice a day in the experimental aquaria and were sampled after exposure to this treatment for one month.

### RNA extraction, reverse transcription, and opsin gene cloning

The red shiners were always sampled between 10:00 AM and 12:00 PM to minimize the effects of circadian rhythms on opsin expression. Fish were anesthetized with 0.025% buffered MS-222 (Ethyl 3-aminobenzoate, methanesulfonic acid salt) solution. After the fish were rendered comatose, body weight and SL of each specimen were measured. An RNeasy mini kit (Cat No./ID: 74104, QIAGEN) was used to isolate total RNA according to the manufacturer’s protocol. The two eyes of each specimen were collected and placed in a 2 ml microcentrifuge tube with stainless steel beads, where the tissue was homogenized by a TissueLyser II (QIAGEN). Total RNA contents and quality were measured by NanoDrop 1000 (Thermo Scientific). Two micrograms of total RNA were reverse transcribed with Super-Script III First-Strand Synthesis SuperMix (Lot. 1372197, Invitrogen); Oligo(dT)20 (Lot. 1352019, Invitrogen) was used as a primer.

The primers from Wang et al. [39] and Chen et al. [40] were used to amplify the opsin genes. PCR amplifications of the opsin genes were performed in a final reaction volume of 25 μL, containing 2 ng cDNA, 6 μmol each of forward and reverse primers, 12.5 μL of Fast-RunTM Advanced Taq Master Mix (ProTech, Taipei, Taiwan), and distilled water. The thermal cycling protocol was as follows: one cycle at 94°C for 4 min; 35 cycles of denaturation at 94°C for 30 sec, 45–60°C for 30 sec, and 72°C for 2 min; one final single extension step at 72°C for 5 min. The PCR products were then purified using a Qiagen purification kit, subcloned into the pGEM-T Easy vector (Promega; Madison, WI), and clones were forward and reversed sequenced using M13 primers. Because of the opsin gene duplication, at least 30 clones for each PCR product were randomly selected and sequenced to obtain all the paralogs of opsin genes. Sequencing was performed using an ABI 3730 version 3.2 analyzer (Applied Biosystems) following protocols of ABI PRISM BigDye Sequencing Kit (PE Applied Biosystems, USA) by Mission Biotech Inc., Taipei, Taiwan. The contig sequences were constructed using the program BioEdit ver. 7.1.9 [41], and the results were identified by BLAST against the National Centre for Biotechnology Information (NCBI) database. The red shiner opsin genes were submitted to NCBI.

### Phylogenetic analysis

Other cyprinid opsin gene sequences were downloaded from NCBI and two genes, VAL-opsin and tmt-opsin, were selected as outgroups [42]. All opsin gene sequences included in the phylogenetic analysis are shown in Table 1. Genes were aligned using the TranslatorX server (http://www.translator.co.uk), which is designed to align protein-coding nucleotide sequences based on their corresponding amino acid translations [43].

**Table 1:** Opsin genes from cyprinid species analyzed in this study.

Scientific Name	tmt-opsin	VAL-opsin	SWS1	SWS2	RH2	LWS	RH1
*Danio rerio*	KT008411	NM_131586	BC060894	NM_131192	NM_131253	NM_001002443	BC045288
					NM_182891	NM_001313715	XM_001336009
					NM_182892		
					NM_131254		
*Candidia barbatus*			EU410458	EU410459	EU410461	EU410457	EU919559
					EU410460		
*Carassius auratus*			D85863	L11864	L11866	L11867	L11863
					L11865	GQ168789	
*Cyprinus carpio*			AB113669	AB113668	AB110603	AB055656	S74449
					AB110602		Z71999
*Zacco pachycephalus*			EU410468	EU410467	EU410463	EU410466	EU919560
					EU410464		
					EU410465		
*Cyprinella lutrensis*			MH013347	MH013348	MH013349	MH000642	MH000641
					MH013350		

Phylogenetic analyses were performed using partitioned Maximum Likelihood (ML). RAxML 8 [44] was used for ML analyses (MLA). Partitions were set with respect to codon position; the GTR+G+I model (with four discrete rate categories) was adopted for each partition. The ML tree was obtained by performing 100 different runs using the default algorithm. The best ML tree was chosen from likelihood scores among suboptimal trees from each run. Nodal support for MLA was bootstrap analysis determined with RAxML [45]; non-parametric bootstrap replications were 1000 with the ML criterion.

### qPCR

The specific primers designed for qPCR were based on the sequences identified from our opsin gene cloning experiments. The amplification efficiency and melting curve of each qPCR primer pair was tested by 5-fold serial dilutions of the templates, with three replicates for each gene and sample. Each qPCR primer pair was adopted only when its amplification efficiency fell between 95% and 105%. Expression of opsin genes and the reference gene was determined by qPCR with an Applied Biosystems StepOnePlus system (Applied Biosystems). Each reaction contained Fast SYBR Green Mastermix (Applied Biosystems), 20 ng of cDNA, and 50 nM of each primer (Table 2) in a final volume of 10 μl. The qPCR reactions were performed in MicroAmp Fast 96-well Reaction Plate (0.1 mL) (Applied Biosystems Ref 4346907) with Optical Adhesive Cover (Applied Biosystems Ref 4360954). The following thermal cycles were performed: one cycle of 50°C for 2 min and 95°C for 10 min, followed by 45 cycles of 95°C for 15 sec and 60°C for 1 min. qPCR products were qualified according to a melting-curve analysis; additionally, representative samples were electrophoresed to verify that only a single product (band) was present. RNA-free water was used as a template in the control reactions to determine nonspecific primer amplification background levels. Three replicates were performed for each cone opsin gene for each specimen.

**Table 2:** Primer sequences used in red shiner opsin gene cloning and qPCR reactions.

	Forward primer (5'-3')	Reverse primer (5'-3')	Source
**Gene cloning**			
Rod opsin			
RH1	TACGTGCCTATGTCCAAYGC	TGCTTGTTCATGCAGATGTAGA	Chen et al. 2008[40]
Cone opsin			
SWS1	ATGGACGCGTGGGCCGTTCAGTTCG	CAGAGAAGTTGTAAATGTGGTGTGG	Wang et al. 2008[39]
SWS2	GGTGTTwCAGCATTCTCGGTGG	TTCACTGCCAGCAGAGTGGTTCTGTC	Wang et al. 2008[39]
RH2	GGCACTGAGGGAAACAACTTCTACATC	GAACATAATCyGTGArAGkTTGACAAG	Wang et al. 2008[39]
	GGCAGAACCATGGmArTTyAAGGC	CACCTCTGTTTTGCTTGTTGAmACTGAG	
LWS	GGGCTATACAACAACCCCAAAAATG	CCTGGCTCAGGATCCTTGCTCTGAG	Wang et al. 2008[39]
**qPCR**			
SWS1	GGCCGAGAAGGAAGTGTCCA	ATCAGCGTTGGCAAACCACA	This study
SWS2	TGCTCTTGTGGACCTGACTGGTA	AACAGAACACGATGGTGGTGAA	This study
RH2A	CCCAYGCTTTGTCAGGAATTGG	GAAGAAGATTACGGTAACCGGAAG	This study
RH2B	CCCACGCTTTGTCAGGAATCGC	GAAGAAGATTATGGTAACCGGGGT	This study
LWS	CAGCTGGTGGCATCATCTTC	AGGCCAATATCTGCTCCAGC	This study
CO1	CCGCCGTACTTCTGCTCCTA	CCTCCTGCTGGGTCAAAGAA	This study

Expression of each opsin gene was calculated using two methods. First, the relative_(hk)_ expression of each opsin gene with respect to the housekeeping gene, cytochrome c oxidase subunit 1 (*CO1*), was calculated according to the following equation:

$$\frac{T_{i}}{T_{co1}}=\frac{\left(1/{\left(1+E_{i}\right)}^{C_{ti}}\right)}{\left(1/{\left(1+E_{co1}\right)}^{C_{tCO1}}\right)}$$

*T*_*i*_*/T*_*CO1*_ is the expression of each opsin gene *i* relative to the expression of *CO1*. *E*_*i*_ is the amplification efficiency for each pair of opsin primers, and *E*_*CO1*_ is the amplification efficiency for *CO1* pair of primer. *C*_*ti*_ is the average critical cycle number for each opsin gene, and *C*_*tCO1*_ is the average critical cycle number for *CO1*. Relative_(hk)_ expression values represent the level of opsin gene expression relative to that of the housekeeping gene, *CO1*.

Second, the proportional expression of the cone opsin genes with respect to the total cone opsin pool was calculated according to the following equation:

$$\frac{T_{i}}{T_{all}}=\frac{\left(1/{\left(1+E_{i}\right)}^{C_{ti}}\right)}{\sum (1/{\left(1+E_{i}\right)}^{C_{ti}})}$$

*T*_*i*_*/T*_*all*_ is the proportional expression for a given opsin gene *i*.

MANOVA (Multivariate analysis of variance) was used to determine if the cone opsin expression profiles differed with turbidity treatment. When MANOVA revealed that cone opsin expression indeed differed by turbidity treatments, the expression of each cone opsin was first examined in the distinct turbidity treatments by Levene’s test and then compared using Tukey’s pairwise comparisons (one-way ANOVA). The statistical tests were performed on R version 3.2.3 (R Foundation for Statistical Computing, Vienna, Austria).

## Results

### Phylogeny of red shiner opsin genes

**Fig 1:**
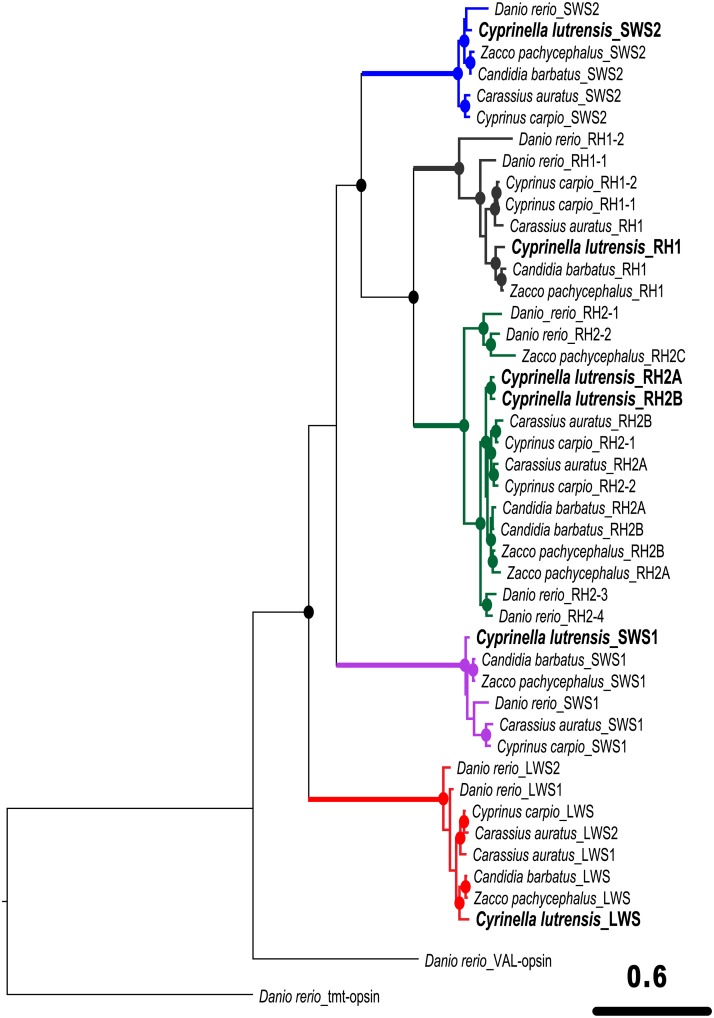
Phylogenetic relationship of the cyprinid opsin genes from partitioned maximum likelihood analysis (three partitions). The opsin gene sequences of the red shiner cloned in this study are in boldface. Solid circles on branch nodes indicate statistically robust nodes with bootstrapping values ≥ 70. Branch color of each opsin family represents its general λ_max_ range. *SWS1*’s is ultraviolet light, *SWS2*’s is blue light, *RH2*’s is green light, and *LWS*’s is red light; also, *RH1*’s is scotopic vision.

A total of 1209 bp were aligned in a dataset of 45 taxa; the dataset contained 1043 variable sites and 846 parsimony-informative sites. Opsin gene cloning revealed that there were six opsin genes expressed in the red shiner retina. These genes include one rod opsin–rhodopsin (*RH1*)–and five cone opsins–one *SWS1* gene, one *SWS2* gene, two *RH2* (*RH2A* and *RH2B*) genes, and one *LWS* gene. In the ML tree (Fig 1), these six opsin genes were clearly clustered with opsin sequences of corresponding types from other cyprinid fishes, with high statistical support (bootstrapping value > 70). Among the visual opsin genes, *LWS* was located at the basal branch as a sister to the group comprising the other four opsin genes. Within the group of four genes, *SWS1* split earlier, *SWS2* was derived later, and *RH1* was a sister to *RH2*. The two *RH2* opsins of the red shiner were determined to be paralogous, similar to *RH2-3* and *RH2-4* in *D*. *rerio* and *RH2A* and *RH2B* in *Zacco pachycephalus*. Conversely, the two *RH2* opsins of *Carassius auratus* and *Cyprinus carpio* are orthologous.

### Light absorbance, ambient light, and cone opsin gene expression

**Fig 2:**
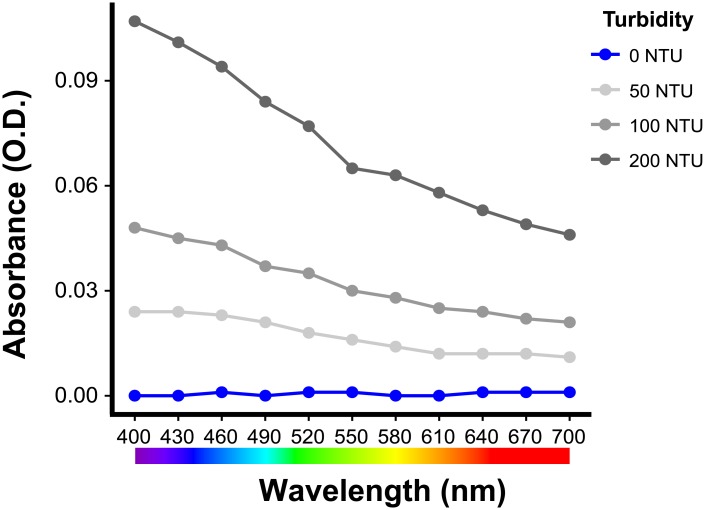
Absorbance spectra from 400 nm to 700 nm of the four turbidity treatments in this study.

**Fig 3:**
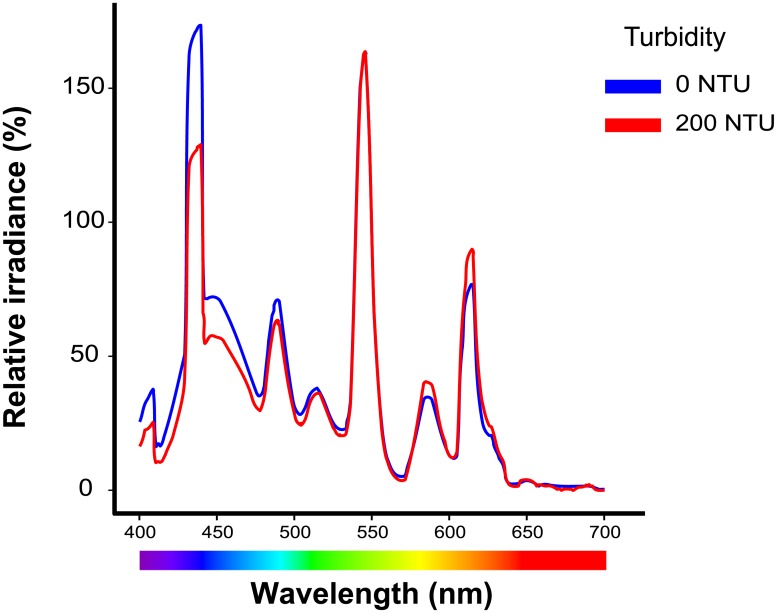
Upward light spectra of the 0 NTU and 200 NTU measured inside experimental aquaria. The relative spectral irradiance normalized by using the intensity at the mid wavelength from 400 nm to 700 nm, 550 nm, is 100%.

The ELISA spectrophotometer measurement revealed that the turbidity treatments (kaolin suspension) absorbed relatively more short than long wavelength light (Fig 2), while the short wavelength was more dominant in the 0 NTU water than in the 200 NTU one (Fig 3). Therefore, in the study, the turbidity treatments resulted in a long wavelength dominant lighting condition.

**Fig 4:**
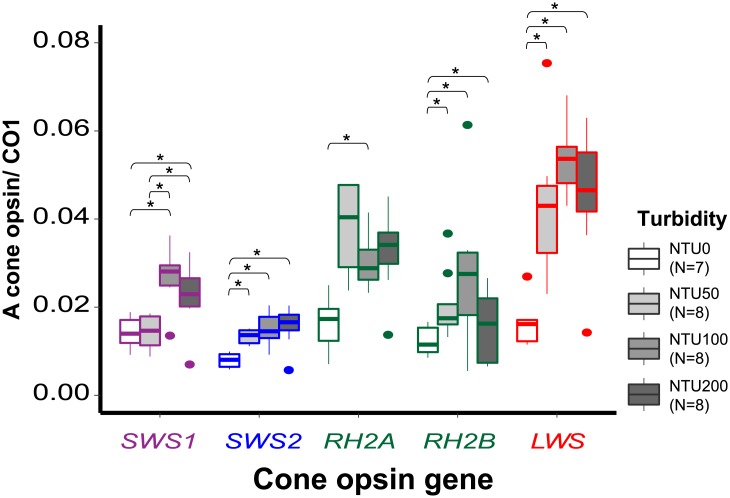
Box plot showing the median (—), 25^th^ and 75^th^ percentiles (box), 95% range (|), and outliers (•) of cone opsin relative expression values for different turbidity treatments. An asterisk indicates *P* < 0.05 (one-way ANOVA, *post hoc* Tukey’s comparisons). The box color of each opsin family represents its general λ_max_ range. *SWS1*’s is ultraviolet light, *SWS2*’s is blue light, *RH2*’s is green light, and *LWS*’s is red light.

**Fig 5:**
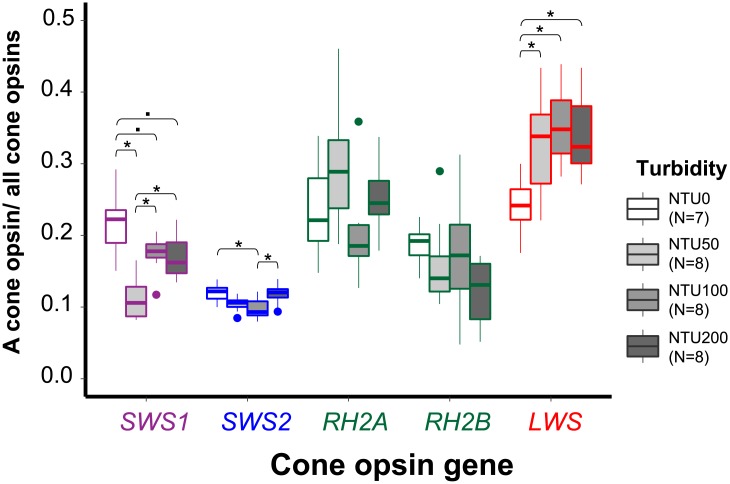
Box plot showing the median (—), 25^th^ and 75^th^ percentiles (box), 95% range (|), and outliers (•) of cone opsin proportional expression values for different turbidity treatments. An asterisk indicates *P* < 0.05 (one-way ANOVA, *post hoc* Tukey’s comparisons) and a dot indicates 0.10 > *P* > 0.05 (one-way ANOVA, *post hoc* Tukey’s comparisons). The color of the boxes of each opsin family represents its general λ_max_ range. *SWS1*’s is ultraviolet light, *SWS2*’s is blue light, *RH2*’s is green light, and *LWS*’s is red light.

The MANOVA analyses revealed that turbidity had an effect on the relative expression of cone opsin genes (Pillai’s Trace: *F*_3, 27_ = 6.729, *P* < 0.01) and the proportional expression (Pillai’s Trace: *F*_3, 27_ = 4.037, *P* < 0.01). The one-way ANOVA test revealed that the relative expression values of each cone opsin were significantly different among different levels of turbidity (*SWS1*: *F*_3, 27_ = 8.862, *P* < 0.01; *SWS2*: *F*_3, 27_ = 8627, *P* < 0.01; *RH2A*: *F*_3, 27_ = 3.220, *P* < 0.05; *RH2B*: *F*_3, 27_ = 8.802, *P* < 0.01; *LWS*: *F*_3, 27_ = 12.837, *P* < 0.01) (Fig 4); however, only the proportional expression values of *SWS1* (*F*_3, 27_ = 12.175, *P* < 0.01), *SWS2* (*F*_3, 27_ = 4.754, *P* < 0.01), and *LWS* (*F*_3, 27_ = 5.712, *P* < 0.01) were significantly different across turbidity levels (Fig 5).

Tukey’s honestly significant difference (HSD) *post hoc* test further demonstrated that the relative expression value of *SWS1* in the 0 NTU environment was significantly lower than those in 100 NTU (*t* = 4.158, *P* < 0.01) and 200 NTU (*t* = 2.760, *P* < 0.05). The relative expression of *SWS2* in 0 NTU was significantly lower than in 50 NTU (*t* = 3.250, *P* < 0.05), NTU100 (*t* = 4.292, *P* < 0.01) and 200 NTU (*t* = 4.593, *P* < 0.01). *RH2A* in 0 NTU was significantly lower than in 100 NTU (*t* = 2.889, *P* < 0.05). *RH2B* in 0 NTU was significantly lower than in 50 NTU (*t* = 4.989, *P* < 0.01), 100 NTU (*t* = 3.167, *P* < 0.05) and 200 NTU (*t* = 3.690, *P* < 0.01). *LWS* in 0 NTU was significantly lower than in 50 NTU (*t* = 4.253, *P* < 0.01), 100 NTU (*t* = 5.912, *P* < 0.01) and 200 NTU (*t* = 4.621, *P* < 0.01). In general, the red shiner expressed more in all five cone opsins in response to increased water turbidity. Moreover, the HSD *post hoc* test demonstrated that the proportional expression of cone opsin *SWS1* in the 0 NTU environment was significantly or marginally higher than those in 50 NTU (*t* = -5.969, *P* < 0.01), 100 NTU (*t* = -2.434, *P* = 0.09), and 200 NTU (*t* = -2.652, *P* = 0.06). The proportional expression of *SWS2* in 0 NTU was significantly higher than that in 100 NTU (*t* = -3.101, *P* = 0.02). *LWS* in 0 NTU was significantly lower than in 50 NTU (*t* = 2.889, *P* = 0.04), 100 NTU (*t* = 3.776, *P* < 0.01), and 200 NTU (*t* = 3.384, *P* = 0.01). Overall, compared to the other opsins, *LWS* became the dominant cone opsin when the red shiners were exposed to turbid water.

## Discussion

Our study sequenced all four cone opsin families; surprisingly, single *RH1*, *SWS1*, *SWS2* and *LWS* genes and two *RH2* genes were amplified from the red shiner. In our phylogenetic analysis (Fig 1), the two *RH2* paralogs of the red shiner were grouped with high statistical support, as were the *RH2-3* and *RH2-4* genes of zebrafish. However, *RH2B* of the *C*. *auratus* was grouped with *RH2-1* of the *C*. *carpio* rather than the *RH2A* gene of the corresponding species. Gene duplications, including tandem duplication and retrotransposition, have increased the number of opsins in ray-finned fish genomes, while the competing processes of pseudogenization, gene truncation and gene loss have all reduced the number of opsins in some species [8, 46]. A gene duplication that occurs before or after speciation defines the paralogs as in-paralogs or out-paralogs, respectively [47]. The phylogenetic analysis in this study supports the notion that *RH2A* and *RH2B* of the red shiner are in-paralogous, but this result may be misleading simply because the *RH2* opsin genes from other closely related species were not included in the analysis. Further studies on the phylogenetic relationship of *RH2* genes from North American leuciscid fishes will help to clarify this issue [48, 49].

An earlier study on genome-wide scanning for opsin genes in ray-finned fish showed that cyprinine fishes may have more than one *LWS* gene and up to five *RH2* genes [8]. There are three possible reasons that some opsin paralogs may not have been cloned in this study. First, the primer selection may have prevented the identification of some opsin paralogs because degenerate primers were used to amplify each opsin family. Second, even if the primers equally amplified each paralog, the paralog expression levels may not have been uniform. To illustrate this idea, the expression levels of *LWS-2* and *LWS-R* are known to be much lower than those of *LWS-1* and *LWS-3* in guppies [25], and the expression level of *SWS2B* is much higher than that of *SWS2A* in *Metriaclima zebra* and *Labeotropheus fuelleborni*, but much reduced in *Oreochromis niloticus* [50]. Therefore, low-expression paralogs might be missed simply due to sampling error in the cloning. Third, the opsin genes were identified from the mRNA extracted from adult retinas; however, fishes often undergo ontogenetic changes in opsin expression [51–53]. This means that opsin paralogs would not be detected by our method if they are not expressed in the red shiner adults. Further work using whole genome sequencing or transcriptome analysis of the retinas from distinct developmental stages may be required to truly uncover the total number of opsins in the red shiner [11, 26, 54–56].

Both the relative and proportional expression levels of cone opsin genes revealed that red shiners are able to adjust cone opsin expression profiles to adapt to different turbidity levels. Therefore, our results supported the hypothesis that the red shiners adults exhibit plasticity in opsin gene expression, which is also observed in the bluefin killifish, African and Nicaraguan cichlids, and some damselfishes [24–28]. In the relative expression analysis, all five cone opsins increased significantly when turbidity values went up; however, in the proportional expression analysis, *SWS1* and *SWS2*, which are responsible for detecting UV to blue light, decreased in response to the higher turbidity, but *LWS*, which is responsible for red light, increased.

Color vision results from the integration of signals from different stimulated cone cells in the retina [7]. The changes in proportional expression of cone opsin genes reflect differences in the proportional abundance of each cone cell type in the retina and/or photopigment density in the cone cells’ outer segments [27, 50, 51], which would bring about variation in color vision. The significant proportional enhancement of *LWS* and reductions of *SWS1* and *SWS2* indicate that the visual spectra of the red shiners shift toward red upon encountering turbid water. Both the absorbance spectra of the turbid treatments and the relative spectra of the 0 NTU and 200 NTU water suggest that turbid treatments in this study had less short wavelength light (Figs 2 and 3). The shift in the visual spectrum toward red is expected to help the visual sensitivity match the ambient light. Moreover, turbid waters often create red light-abundant surroundings [57–59], so it is not surprising that the guppy also has a higher proportional and relative *LWS* opsin expression when it is reared in turbid water [25]. Besides matching the ambient light of turbid waters, *LWS* is very important for motion detection since it is expressed in L-class cone cells [60–62]. The three-spined stickledback (*Gasterosteus aculeatus*) was shown to suffer from search inefficiency in humic water, possibly due to limited plasticity in opsin expression [63, 64]. Hendry [65] asserted that plasticity in an important fitness-related trait could help species colonize; therefore, adult opsin expression plasticity may enable red shiners to successfully adapt to new aquatic environments and thrive in the newly invaded areas [66–69].

All shiners are phylogenetically closely related and many are sympatric; moreover, historical and current genetic introgressions have been detected in shiners [48, 70]. Reproductive behavior is an important pre-mating isolating mechanism to inhibit hybridization among shiners. Indeed, Blum et al. [71] demonstrated that, although the postzygotic reproductive isolation between the red shiner and the blacktail shiner (*C*. *venusta*) was incomplete, the females of these species were more responsive to conspecific mates. Interestingly, both laboratory experiments and field data demonstrated that turbidity weakened the strength of the behavioral isolation between these two shiners and hence made hybridization possible [67, 72].

According to the model presented by Mitchem et al. [73], color perception relies on the spectral sensitivity of the viewer, side-welling irradiance, and reflectance of the object. Turbid habitats could degenerate the color perception of the red and/or blacktail shiners, inhibiting their ability to choose to mate with conspecifics. Moreover, turbidity may also affect breeding behavior [74, 75], so that blacktail and red shiners hybridize in turbid conditions due to changes in both visual perception and behavior [72].

Finally, the loss of opsin expression plasticity in adults supports the idea that it is costly to remodel a phenotype once it is developed [25, 63, 76]. Thus, in order to maintain plasticity in the adult stage, selection has to at least neutralize these prohibitive costs and genetic drift. Stieb et al. [29] used damselfishes as a model to show that plasticity in opsin expression is possibly related to ecological differences in visual tasks. The range of depth distribution might correlate to the degree of plasticity, or it may be under phylogenetic constraints. Nandamuri et al. [28] suggested that the geographic history of Lake Malawi, fluctuating between being a “green” and “blue” lake [77], generates a selective force to maintain plasticity. The red shiner’s wide distribution range encompasses a large variety of aquatic habitats [48], which could serve as a similar force to maintain plasticity in opsin gene expression; moreover, high genetic diversity in the species and gene flow among conspecific and heterospecific lineages may suppress the influence of genetic drift [69, 78, 79]. Further studies on the demographic and biogeographic history of the red shiner, in addition to studies on opsin expression plasticity in other closely related leuciscid fishes, would help to answer whether the geographic history of North America and/or phylogenetic constraints help preserve plasticity.

## Supporting information

S1 Table qPCR results for five cone opsins. The original data of cone opsins relative and proportional expression values for different turbidity treatments.
(CSV)

S2 Table Upward light spectra of the 0 NTU and 200 NTU measured inside experimental aquaria. The original data of light spectra of the 0 NTU and 200 NTU.
(CSV)

S3 Table Absorbance spectra from 400 nm to 700 nm of the four turbidity treatments. The original data of absorbance spectra measured by VersaMax ELISA Plate Reader.
(CSV)
